# Bexarotene signaling in human B and T lymphocytes induces gut-homing receptor expression

**DOI:** 10.3389/fimmu.2025.1664199

**Published:** 2025-10-08

**Authors:** Sina Kaiser, Ina Suhrkamp, Jinru He, Charlotte Helf, Christian D. Sadik, Michael Weichenthal, Guido Heine

**Affiliations:** ^1^ Department of Dermatology, Venereology, and Allergy, University Hospital Schleswig-Holstein, Kiel, Germany; ^2^ Zoological Institute, Kiel University, Kiel, Germany; ^3^ Department of Dermatology, Venereology, and Allergy, University of Lübeck, Lübeck, Germany

**Keywords:** B cells, bexarotene, homing, lymphocytes, retinoid, rexinoid, T cells

## Abstract

**Background:**

Retinoic acid (RA) receptors (RARs) in human lymphocytes modulate the humoral and intestinal immune response by regulating target genes, including CD38, TGM2, and gut-homing markers. The impact of retinoid X receptors (RXRs) on this process is elusive.

**Objective:**

To determine the impact of the RXR ligand bexarotene (BXR) on the activation and differentiation of human B and T lymphocytes.

**Methods:**

*In vitro* BXR stimulation of human CD19^+^ B cells and CD4^+^ T helper cells was investigated regarding retinoid target gene expression using qPCR and flow cytometry and validated in peripheral B and T lymphocytes of patients with cutaneous T-cell lymphoma (CTCL) with and without BXR treatment.

**Results:**

BXR induced the canonical retinoid target gene *CD38* in B cells and T cells (sixfold and threefold, respectively). BXR increased CD38 surface protein expression on B cells twofold and plasmablast differentiation threefold. The frequency of the gut-homing receptors CCR9 and integrin β7 was doubled on T and B cells after BXR stimulation, while cutaneous leucocyte-associated antigen (CLA) expression was decreased in B cells. Under BXR treatment, a reduced frequency of cells with these gut-homing receptors was observed in the blood of CTCL patients regarding memory T cells (mean off: 1.9%; on: 0.6%) and B cells (mean off: 5.7%, on: 4%).

**Conclusion:**

BXR via RXRs directly targets B and T lymphocytes, inducing retinoid target gene expression, including gut-homing receptors.

## Introduction

Vitamin A influences the humoral immune response. Individuals with vitamin A deficiency are susceptible to fatal infections due to a strongly impaired humoral immune response (1). Thus, the World Health Organization recommends vitamin A supplementation before vaccinations in high-risk regions (2). Retinoids are biologically active vitamin A metabolites and activate nuclear retinoic acid receptors (RARs) or retinoid X receptors (RXRs) (3). The physiological agonist 9-*cis*-retinoic acid (9cRA), which acts as a pan-agonist binding to all RARs and RXRs (4), is used for the treatment of hand eczema (alitretinoin), while all-*trans*-retinoic acid (ATRA), binding to RARs, is used to treat acne vulgaris (tretinoin). The synthetic rexinoid bexarotene (BXR) selectively binds to RXRs. As BXR sometimes induces hypertriglyceridemia, other less triglycemic derivatives are also being investigated in cancer research (5). Other rexinoids, they induce less hypertriglyceridemia. Therefore, less triglycemic derivatives, are also being investigated in cancer research (6–8). The biological profile of vitamin A metabolites results from specific DNA binding motifs of respective receptor dimers, e.g., the DR-5 motif, referring to 5 spacer nucleotides between the direct repeats of RARs and RXRs or the DR-1 motif of two RXRs spaced by one nucleotide (9). The immune functions of RARα, the dominant receptor in T and B lymphocytes, have been studied in most detail. In humans and mice, similar data were obtained studying ATRA, *RARA* overexpression (10), or dominant negative RARα (11) in line with a high phylogenetic conservation. 9cRA signaling, most likely via RARα, results in the upregulation of the canonical target genes *CD38* and *TGM2* (12), B-cell differentiation toward plasma cells (12), and isotype class switch to immunoglobulin A (IgA) at the expense of IgE in allergy (13). In addition, ATRA induces the expression of gut-homing markers in human T cells *in vitro* (14), similar to gut dendritic cells producing RA and inducing gut-homing marker expression on murine naïve B cells and human spleen-derived naïve or memory B cells (15). However, little is known about the immune functions of BXR, beyond murine T cells (16) and CTCL cell lines (17).

In this study, we investigated whether BXR acts directly on human B and T lymphocytes *in vitro*, followed by *ex vivo* analyses of CTCL patients with or without BXR treatment.

## Materials and methods

### Cells and culture conditions

Buffy coats were obtained from the Institute of Transfusion Medicine at the University Hospital Schleswig-Holstein, Kiel. CTCL patients with planned BXR treatment (n = 4, **Supplementary Table 1**) were recruited at the Department of Dermatology, Venereology and Allergy, University Hospital Schleswig-Holstein, Kiel.

Peripheral blood mononuclear cells (PBMCs) were isolated using Polysucrose 400 gradient (BioClot GmbH, Aidenbach, Germany) according to the manufacturer’s instructions. *In vitro* cultures of PBMCs were performed using 5 × 10^5^ cells/mL in Iscove’s Modified Dulbecco’s Medium (IMDM; Gibco, Thermo Fisher Scientific, Waltham, MA, USA), 10% fetal calf serum (FCS; Capricorn Scientific, Ebsdorfergrund, Germany), 0.1 mM non-essential amino acids (Sigma-Aldrich, St Louis, MO, USA), 2 mM GlutaMAX™ (Gibco), and transferrin and insulin (both Sigma-Aldrich, 5 µg/mL) (12). B cells were activated with CD40 ligand (CD40L, 1 µg/mL), IL-4 (5 ng/mL), IL-10 (50 ng/mL), IL-21 (50 ng/mL) (all Miltenyi Biotec, Bergisch Gladbach, Germany), and CpG 2006 (3 µg/mL, TIB Molbiol Syntheselabor GmbH, Berlin, Germany). T cells were activated with anti-CD3 (1 µg/mL, OKT3) and anti-CD28 (0.5 µg/mL, 15E8, both Miltenyi Biotec). To analyze the effect of 9cRA and BXR using flow cytometry, the cells were stimulated with 0.1 µM 9cRA (Enzo Life Sciences, Farmingdale, NY, USA) or 1 µM BXR (Sigma-Aldrich), unless indicated otherwise, for 4 days, in some experiments with 3 h preincubation of 100-fold excess of the pan-RAR inhibitor AGN194310 (RARi; Sigma-Aldrich; 1 or 10 µM). Secreted immunoglobulins were measured in the supernatant on day 4. The proliferation was analyzed using carboxyfluorescein succinimidyl ester (CFSE) staining (BioLegend) according to the manufacturer’s instructions before stimulating the cells as described above. For transcriptional analysis, CD19^+^ B cells or CD4^+^ T cells were purified by positive magnetic cell sorting according to the manufacturer’s instructions (Miltenyi Biotec); 1.5 × 10^6^ B or T cells/mL were stimulated for 2 days as described above.

### Flow cytometry

Patient-derived PBMCs were analyzed directly *ex vivo*; 5 × 10^5^ stimulated B or T lymphocytes were analyzed after 4–11 days of cultivation. Kinetic studies have unraveled day 4 as the earliest time point showing retinoid-mediated effects with few secondary effects of differentiation (data not shown), with the following markers of retinoid signaling: CLA-PE (HECA-452), CD49d (integrin α4)-PE-Cyanine7 (9F10), chemokine receptor 9 (CCR9)-APC (L053E8), CD38-Brilliant Violet 650 (HB-7, all Biolegend, San Diego, CA, USA), transglutaminase 2 (TGM2)-AlexaFluor 700 (TGM2/419, Novus Biologicals, Centennial, CO, USA), and integrin β7-BV421 (FIB504, BD Biosciences, Franklin Lakes, NJ, USA). Additionally, the B-cell panel was applied using CD27-FITC (M-T271), IgD-APC-Vio 770 (REA740), CD3-VioGreen (REA613), CD14-VioGreen (REA599, all Miltenyi Biotec), and CD19-Brilliant Violet 785 (HIB19, BioLegend). T cells were stained with CD3-FITC (OKT3), CD4-PerCP-Cyanine5.5 (OKT4), CD197 (CCR7)-APC/Cyanine7 (G043H7), and CD45RO-Brilliant Violet 510 (UCHL1, all BioLegend). For the proliferation and viability analyses, the PBMCs were stained with CCR9-APC (L053E8), CD197-APC/Cyanine7 (G043H7), CD4-PE (OKT4), CD3-AlexaFluor 700 (OKT3), CD45RO-Brilliant Violet 421 (UCHL1), CD27-Brilliant Violet 510 (O323), IgD-Brilliant Violet 605, CD38-Brilliant Violet 650 (HB-7), and CD19-Brilliant Violet 785 (HIB19, all BioLegend). Five minutes before the measurement, 7-amino-actinomycin D (7-AAD, BioLegend) was added to analyze viability. The number of proliferating cells was calculated by multiplying the cell number according to the respective division (division 1 × 1, division 2 × 2, division 3 × 4, and division 4 × 8). Flow cytometry analysis was conducted on a CytoFLEX flow cytometer using the CytExpert software (Beckman Coulter Life Sciences, Brea, CA, USA). The data were analyzed using the FlowJo v10 software (BD Biosciences).

### RNA isolation and quantitative PCR

RNA was isolated (NucleoSpin RNA kit, Macherey-Nagel, Düren, Germany) and quantified using a NanoDrop spectrophotometer (Thermo Fisher Scientific, Waltham, MA, USA), and equal amounts of RNA from each sample were reverse transcribed (TaqMan Reverse Transcription Reagents kit, Applied Biosystems, Thermo Fisher Scientific, Waltham, MA, USA), as described previously (12). Quantitative PCR of CD38 and TGM2 was performed on QuantStudio3 (TB Green Premix Ex Taq II kit, Takara Bio, Kusatsu, Shiga, Japan) and normalized to hypoxanthine phosphoribosyltransferase (HPRT) expression. The oligonucleotides used were as follows: HPRT for ATC AGA CTG AAG AGC TAT TGT AAT GAC CA and rev TGG CTT ATA TCC AAC ACT TCG TG (18), CD38 for TGG CGC GAT GCG TCA AGT ACA and rev GGG TGA ACA TGT CCC GCT GGA (12), TGM2 for GAG CAG AAG ACG GTG GAG A and rev AAG CCC TTC ACA GCC TTC A, RXRA for TGC TTC GTG TAA GCA AGT ACA TAA G and rev CTC TTT ATG GAT CTG TCA TCC TCT C, RXRB for CCA GAG TCT CTT TTT ACA CTT CAC C and rev TCT TAG TCA ACC TGG GAA AGT ACA G, and RXRG for GAT CTA GAG GCA GAT TCC TGA CTA A and rev CAT GTT TAC TCG TCA GTT CAT GTT C (19) (all from Eurofins Scientific SE, Luxembourg; displayed in 5′–3′ format).

### Immunoglobulin quantification

Secreted IgG1, IgG2, IgG3, IgG4, IgA, and IgM were quantified from the supernatant of stimulated cells after 4 days using a bead-based multiplex fluorescence immunoassay (LEGENDplex human immunoglobulin isotyping panel, BioLegend).

### Promoter analysis

The 10-kb 5′ UTR regions of candidate genes were scanned for human RAR and RXR binding sites using related motif profiles downloaded from the JASPAR database (10th release, 20) by FIMO (v5.5.7, 21). The first exons were used as negative controls, and the reported DR-5 in the first intron of CD38 (22) was used as a positive control for quality filtering. All genomic sequences were downloaded from the UCSC genome browser (GRCh38/hg38). The identified binding sites were filtered using p-value thresholds and classified using the spacing patterns.

### Statistical analyses

Statistical analyses were performed using GraphPad Prism (version 10.3.1). Normal distribution was analyzed using the Shapiro–Wilk test. The group comparisons were performed using a paired t-test or a one-sample t-test. The asterisks were used for group comparisons to the dimethyl sulfoxide (DMSO) control (stimulated without retinoids, normalized to 1) to indicate statistical significance (*p < 0.05; **p < 0.01; ***p < 0.001), and the hashtags were used for the comparison of RARi and 9cRA or BXR compared to only 9cRA (positive control) or BXR (0.1 µM) accordingly.

### Ethical statement

Written informed consent was obtained from all participants. All procedures were approved by the local ethics committee (D519/20) following the Declaration of Helsinki.

## Results

### Bexarotene increases *CD38* gene expression in B and T lymphocytes

To analyze whether BXR activates retinoid receptors in lymphocytes directly, the expression of typical retinoid target genes *CD38* and *TGM2* (12) was quantified using qPCR from purified CD19^+^ B and CD4^+^ T lymphocytes after stimulation with BXR or 9cRA in the presence or absence of the pan-RAR antagonist RARi. Computational prediction models suggest that classical retinoid binding DR-5 motifs, as well as rexinoid binding DR-1 motifs, are present in the *CD38* and *TGM2* promoter regions (**Supplementary Table 2**). Also, human PBMCs express RARs and RXRs (23). More specifically, *RXRA* and *RXRB* are expressed in human B cells, while the expression of *RXRG* is very low in memory and plasma cells alike (24). In this line, *RXRA* and *RXRB* mRNAs were expressed in activated B cells, which were not altered by stimulation with 9cRA or BXR (**Supplementary Figure 1** and not shown). Similarly, also in stimulated T cells, *RXRA* and *RXRB* were expressed, which were not altered by the addition of 9cRA or BXR (**Supplementary Figure 1** and not shown), in line with previous findings (25, 26).

**Figure 1:**
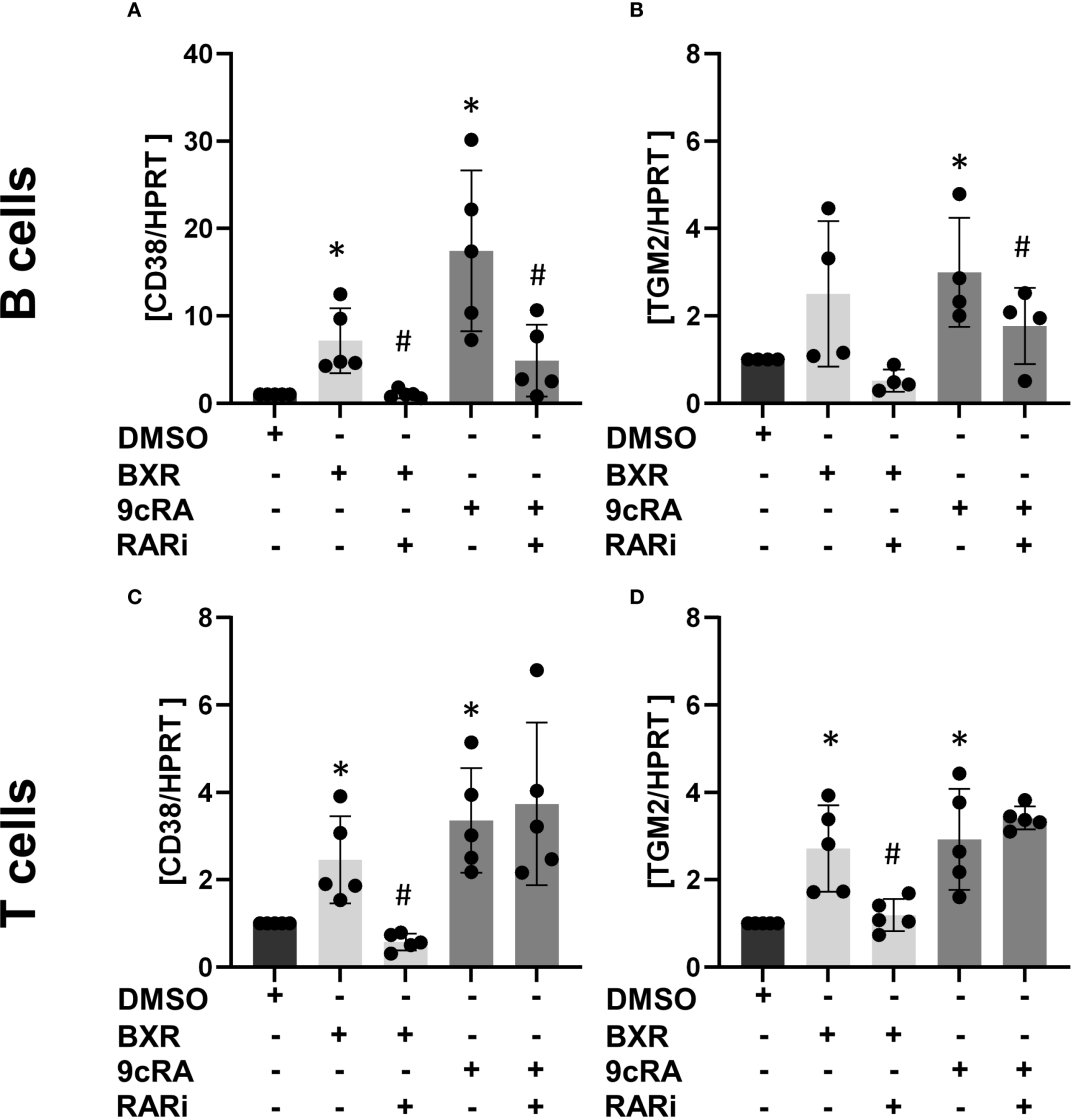
Bexarotene induces *CD38* and *TGM2* transcription. Isolated B cells (n = at least 4; **(A, B)** and T cells (n = 5; **(C, D)** were stimulated with BXR or 9cRA as well as a combination of 9cRA or BXR and RARi (all 1 µM) for 2 days. The transcription of the retinoid response genes CD38 **(A, C)** and TGM2 **(B, D)** was measured using qPCR. The data were normalized to HPRT and then to DMSO stimulation and analyzed using one-sample t-test (*p < 0.05) compared to DMSO control or the RARi combination with 9cRA or BXR by paired t-test compared to 9cRA or BXR stimulation, respectively (#p < 0.05). Each data point represents one healthy donor, and error bars indicate mean + SD. BXR, bexarotene; 9cRA, 9-*cis*-retinoic acid; RARi, retinoic acid receptor inhibitor AGN194310.

The data showed a low *CD38* gene expression on B cells with B-cell stimulation, which increased 17-fold by 9cRA (p = 0.02; **Figure 1A**). In T cells, the expression increased 3.4-fold (p = 0.01; **Figure 1C**). Conversely, the addition of RARi abolished the 9cRA-induced CD38 induction in B cells (p = 0.01, 27% of 9cRA stimulation; **Figure 1A**). In response to BXR, CD38 expression was significantly increased in B cells (7.2-fold, p = 0.02; **Figure 1A**) and T cells (2.5-fold, p = 0.03; **Figure 1C**), which was inhibited by RARi in B cells (13.9% of BXR, p = 0.01) and T cells (23.2% of BXR, p = 0.02). TGM2 was expressed at low levels in stimulated lymphocytes. It was upregulated by 9cRA in B cells (threefold, p = 0.049; **Figure 1B**) as well as T cells (2.9-fold, p = 0.02; **Figure 1D**). Also, BXR upregulated TGM2 in T cells (2.7-fold, p = 0.02; **Figure 1D**), which was inhibited by RARi (44.4% of BXR stimulation, p = 0.02). Thus, these data demonstrated that BXR induces retinoid response genes in lymphocytes.

### Increased gut-homing marker expression on human peripheral B cells by bexarotene

**Figure 2:**
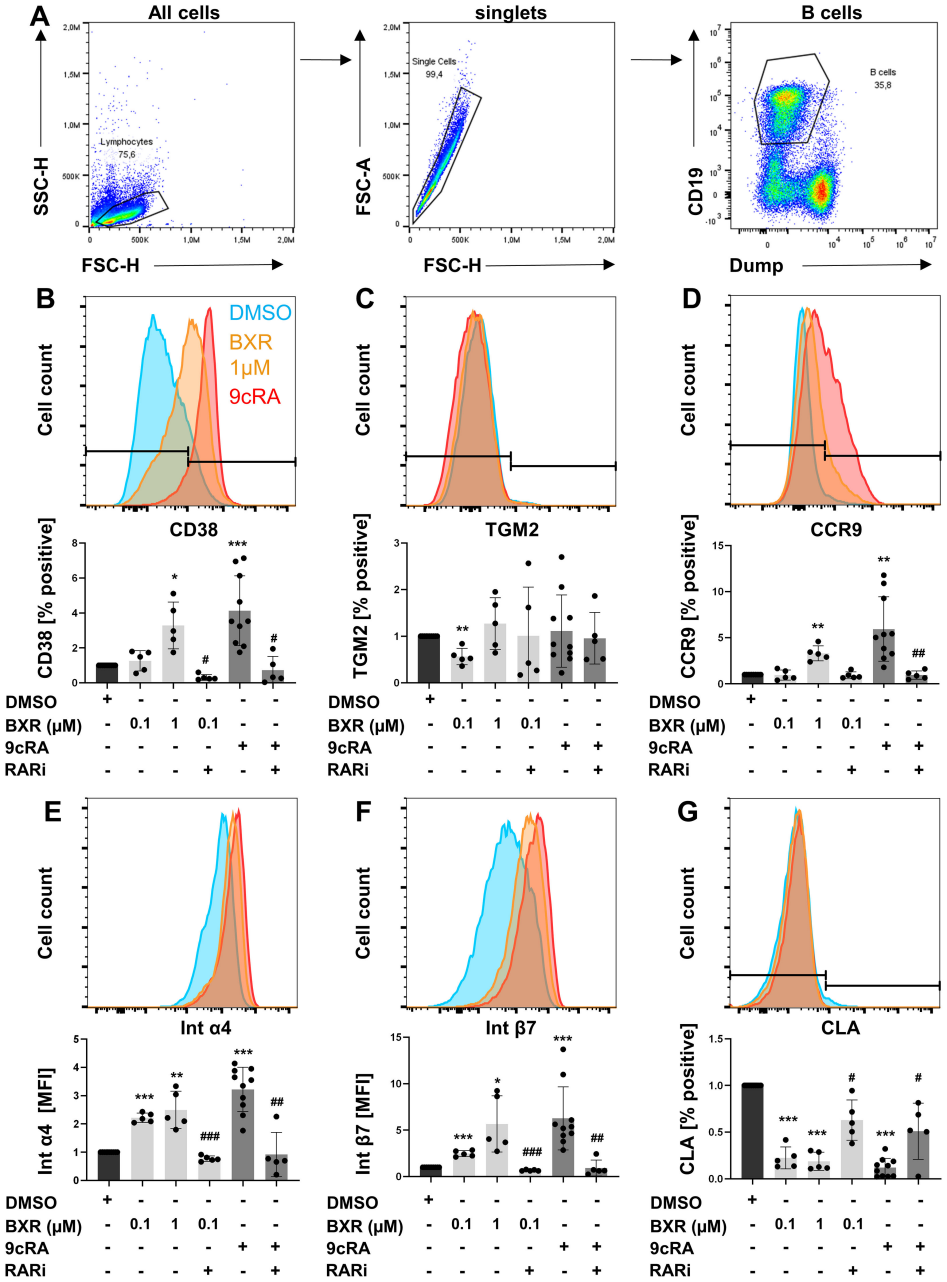
Increased gut-homing marker expression on B cells after retinoid stimulation. PBMCs (n = at least 5) were stimulated with CD40L, IL-21, IL-10, IL-4, and CpG 2006 in presence and absence of BXR (0.1 and 1 µM) or 9cRA (0.1 µM) with and without RARi (10 µM) for 4 days, and the expression of homing markers was analyzed. **(A)** Gating strategy of B cells. Histogram overlays of **(B)** CD38, **(C)** TGM2, **(D)** CCR9, **(E)** integrin α4, **(F)** integrin β7, and **(G)** CLA from a representative donor and respective bar diagrams of surface expression shown as percentage positive of B cells or MFI. The data were normalized to the DMSO control, tested for normality by Shapiro–Wilk test, and analyzed using one-sample t-test compared to DMSO stimulation (*p < 0.05. **p < 0.01, and ***p < 0.001) or the RARi combination with 9cRA or BXR analyzed using paired t-test (#p < 0.05, ##p < 0.01, and ###p < 0.001) compared to 9cRA or 0.1 µM BXR stimulation, respectively. Each data point represents one healthy donor, and error bars indicate mean + SD. PBMCs, peripheral blood mononuclear cells; BXR, bexarotene; RARi, retinoic acid receptor inhibitor AGN194310; CLA, cutaneous leucocyte-associated antigen; MFI, mean fluorescence intensity; DMSO, dimethyl sulfoxide; 9cRA, 9-*cis*-retinoic acid.

To confirm retinoid signaling by BXR at the protein level, human B cells (**Figure 2A**) were stimulated with BXR, and the surface expression of typical retinoid-induced proteins was determined using flow cytometry. The frequency of CD38^+^ B cells was 26% of B cells after stimulation with CD40 ligand, CpG, IL-21, IL-10, and IL-4. The addition of 9cRA increased CD38 expression to 87% of B cells (p < 0.001), which was reduced by RARi to 17%. Of note, BXR upregulated CD38 expression to up to 65% of B cells (p = 0.02), which was reduced to 11% by RARi (p = 0.03; **Figure 2B**). In B cells, neither 9cRA nor 1 µM BXR changed TGM2 expression at the protein level (**Figure 2C**). Of note, the promoter regions of several retinoid-induced genes, including CCR9, integrin α4, integrin β7, and CLA, also contained DR-5 as well as DR-1 motifs (**Supplementary Table 2**). Accordingly, 9cRA enhanced the expression of CCR9 (sixfold, p = 0.002; **Figure 2D**), integrin α4 (threefold, p < 0.001; **Figure 2E**), and integrin β7 (sixfold, p = 0.001; **Figure 2F**). This was significantly inhibited by RARi for these surface proteins (CCR9, 26% of 9cRA stimulation, p = 0.006; integrin α4, 26%, p = 0.002; integrin β7, 19%, p = 0.001; **Figures 2D–F**). In the presence of BXR, these gut-homing receptors were also upregulated with a comparable pattern but to a lesser extent. In detail, CCR9 was significantly induced only by 1 µM BXR (threefold, p = 0.003), while integrin α4 (up to 2.5-fold, p < 0.001 and p = 0.007) and integrin β7 (up to 5.7-fold, p = 0.001 and p = 0.03; **Figures 2D–F**) were already significantly induced at 0.1 µM, which was reduced by RARi regarding integrin α4 to 35% and integrin β7 to 27% of the 0.1 µM BXR stimulation (both p < 0.001). The frequencies of CLA^+^ B cells were significantly reduced from 6% to 0.4% and 0.5% by both 9cRA and BXR (both p < 0.001), respectively, and this decrease was inhibited by RARi for 9cRA (4.7%, p = 0.02) and 0.1 µM BXR (5.5%, p = 0.02; **Figure 2G**). In sum, human B cells respond to BXR and 9cRA, both of which induce gut-homing receptors.

### Increased gut-homing marker expression on human peripheral T cells by bexarotene

**Figure 3:**
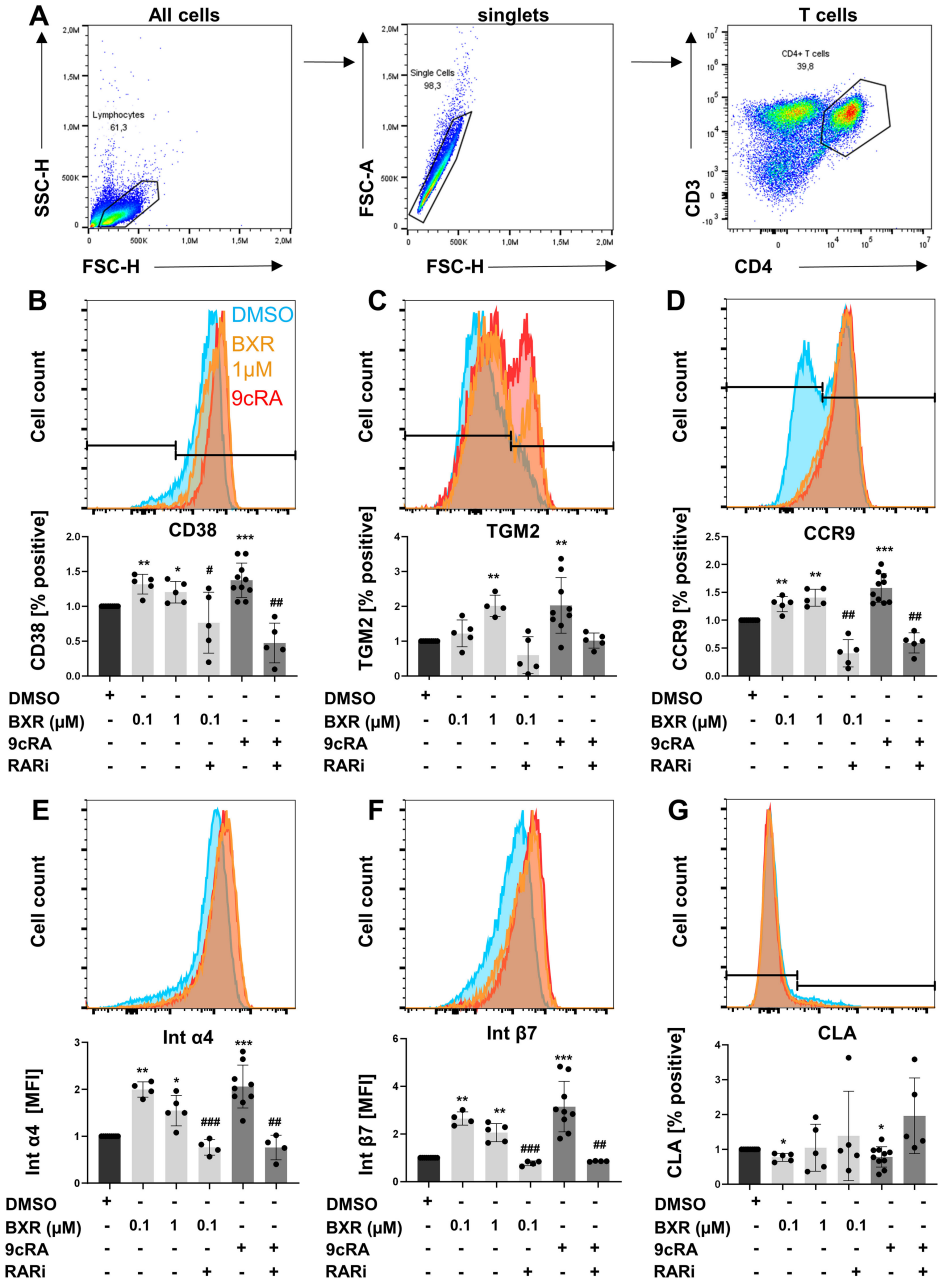
Increased gut-homing marker expression on T cells after retinoid stimulation. PBMCs (n = at least 5) were stimulated with anti-CD3 and anti-CD28 in presence and absence of BXR (0.1 µM and 1 µM) or 9cRA (0.1 µM) with and without RARi (10 µM) for 4 days, and the expression of homing markers was analyzed. Gating strategy of T cells **(A)**. Histogram overlays of **(B)** CD38, **(C)** TGM2, **(D)** CCR9, **(E)** integrin α4, **(F)** integrin β7, and **(G)** CLA from a representative donor and respective bar diagrams of surface expression shown as percentage positive of B cells or MFI. The data were normalized to the DMSO control, tested for normality by Shapiro–Wilk test, and analyzed using one-sample t-test compared to DMSO stimulation (*p < 0.05, **p < 0.01, and ***p < 0.001) or the RARi combination with 9cRA or BXR analyzed using paired t-test (#p < 0.05, ##p < 0.01, and ###p < 0.001) compared to 9cRA or 0.1 µM BXR stimulation, respectively. Each data point represents one healthy donor, and error bars indicate mean + SD. PBMCs, peripheral blood mononuclear cells; BXR, bexarotene; 9cRA, 9-*cis*-retinoic acid; RARi, retinoic acid receptor inhibitor AGN194310; CLA, cutaneous leucocyte-associated antigen; MFI, mean fluorescence intensity; DMSO, dimethyl sulfoxide.

To investigate the impact of BXR signaling in human T helper cells (**Figure 3A**), the expression of retinoid-induced surface molecules on human CD4^+^ T cells activated *in vitro* was analyzed. The data showed that most T cells expressed CD38 after CD3/CD28 stimulation (64%), which was increased by 9cRA stimulation (84%, p = 0.001) and inhibited by RARi (22%, p = 0.003; **Figure 3B**). Of note, BXR enhanced the frequency of CD38^+^ T cells to 92% (p = 0.04; **Figure 3B**) and was inhibited by RARi (37%, p = 0.04). Similar to the mRNA regulation of the canonical target gene, 9cRA additionally enhanced TGM2 expression by T cells, similar to BXR stimulation (both twofold, p = 0.005 and p = 0.007; **Figure 3C**). The gut-homing markers were induced by 9cRA, resulting in enhanced expression of CCR9, integrin α4, and integrin β7 (1.6-, 2.1-, and 3.1-fold, respectively, all p < 0.001; **Figures 3D–F**), which accordingly were reduced by RARi (CCR9, 40% of 9cRA, p = 0.006; integrin α4, 29%, p = 0.007; integrin β7; 20%, p = 0.006). Of note, also the rexinoid BXR increased the gut-homing marker expression similar to 9cRA. The percentage of CCR9^+^ T cells was increased up to 1.4-fold (p < 0.004) and was inhibited by RARi (32% of 0.1 µM BXR, p = 0.001). Integrin α4 (up to twofold, p = 0.001) and integrin β7 (up to 2.6-fold, p = 0.001) were enhanced by BXR stimulation as well and were both inhibited by RARi (38% and 30% of 0.1 µM BXR, respectively, both p < 0.001). In contrast, 9cRA decreased CLA expression to 78% of DMSO-stimulated cells (p = 0.05, **Figure 3G**), similar to BXR (77%, p = 0.01). Taken together, BXR regulated gut-homing receptor surface expression on human peripheral T cells similar to 9cRA.

### Bexarotene promotes differentiation into antibody-secreting cells

**Figure 4:**
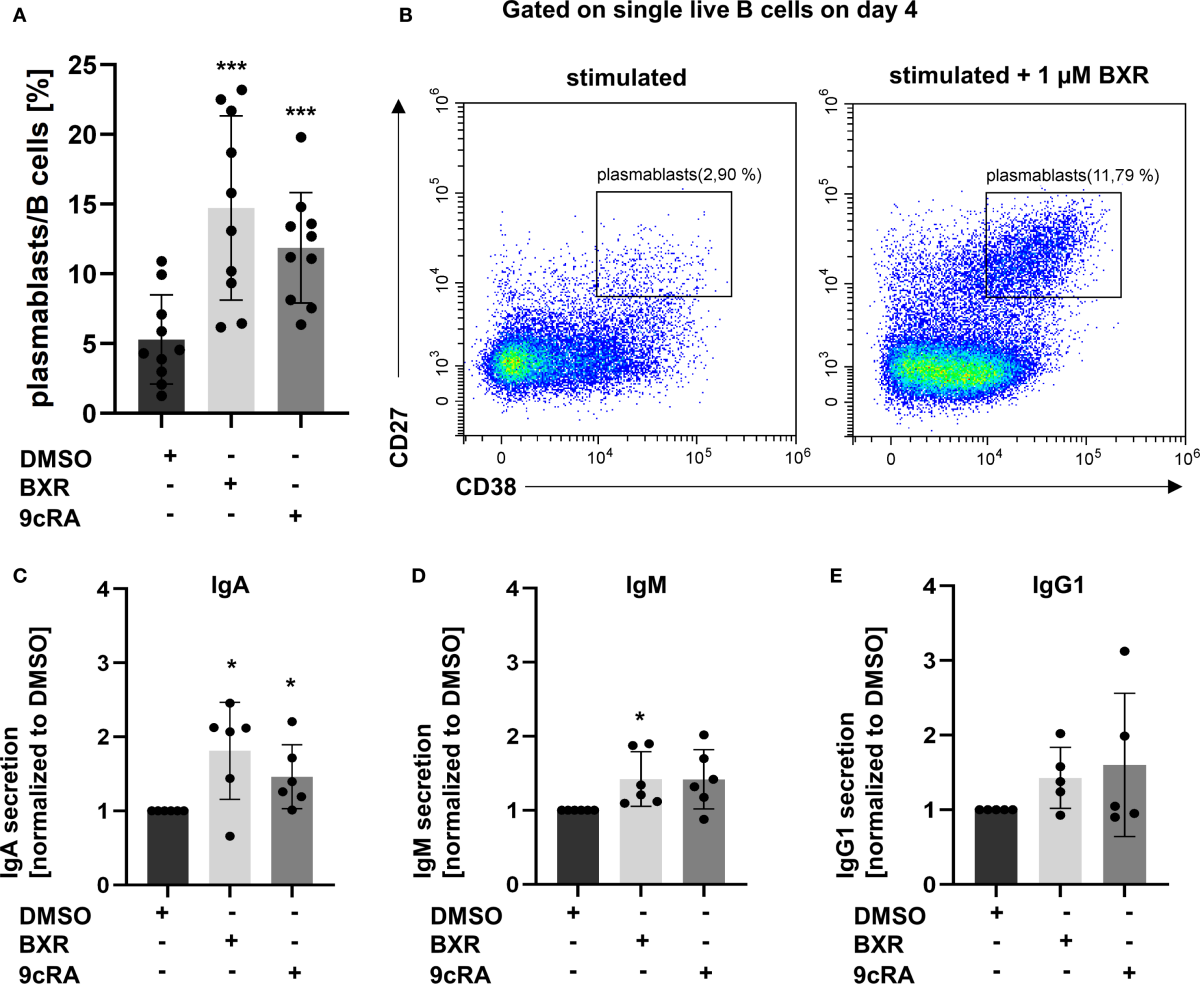
Bexarotene stimulation increases plasmablasts as well as IgA and IgM antibody secretion. PBMCs (n = at least 5) were stimulated with CD40L, IL-21, IL-10, IL-4, and CpG 2006 and BXR (1 µM) or 9cRA (0.1 µM) for 4 days. Gated on single live B cells. **(A)** Percentage of plasmablasts. **(B)** Representative plot of plasmablasts with BXR stimulation compared to DMSO control. The supernatant was analyzed for secreted antibodies of **(C)** IgA, **(D)** IgM, and **(E)** IgG1 isotypes. The data were normalized to DMSO and analyzed using one-sample t-test (*p < 0.05, **p < 0.01, and ***p < 0.001) compared to DMSO control. Each data point represents one healthy donor, and error bars indicate mean + SD. PBMCs, peripheral blood mononuclear cells; BXR, bexarotene; 9cRA, 9-*cis*-retinoic acid; DMSO, dimethyl sulfoxide.

To analyze if BXR promotes IgA plasmablast formation, as known for the retinoids 9cRA and ATRA, stimulated B cells on day 4 were analyzed using flow cytometry. The differentiation to CD27^hi^ CD38^hi^ plasmablasts was detected, and the secreted Ig was quantified using a fluorescence immunoassay. In the presence of 9cRA, the frequencies of plasmablasts were increased 2.8-fold compared to the B cell-stimulated control (p < 0.005; **Figure 4A**). Similarly, BXR stimulation increased plasmablast differentiation 2.6-fold compared with control (p = 0.006; **Figures 4A, B**). Accordingly, the concentrations of secreted IgA were increased by 9cRA and BXR compared to the stimulated control (1.5-fold, p = 0.047, and 1.8-fold, p = 0.03, respectively; **Figure 4C**). For IgM, the concentration was increased 1.4-fold for BXR (p = 0.04; **Figure 4D**). The secreted concentrations of IgG1 (**Figure 4E**), IgG3, and IgG4 were not significantly altered by 9cRA or BXR, and IgG2 was below the detection threshold on day 4 (not shown).

A proposed mechanism of action of BXR in CTCL is apoptosis (27). Our data showed no impact of BXR or 9cRA on the viability of B or T cells on day 4 (**Supplementary Figure 2**). In this matter, ATRA was shown to promote B-cell proliferation (28). Here, CFSE-labelled, activated lymphocytes were analyzed on day 4. BXR and 9cRA increased the proliferation of B cells (**Supplementary Figure 3A, B**) and T cells (**Supplementary Figures 3C–E**). The viability of highly proliferating and resting cells was comparable with or without BXR or 9cRA (data not shown). Thus, the rexinoid BXR enhances plasmablast differentiation into IgA-secreting cells without an impact on the viability.

### Bexarotene reduces the frequency of CCR9^+^ integrin β7^+^ memory B and T lymphocytes in the blood

**Figure 5:**
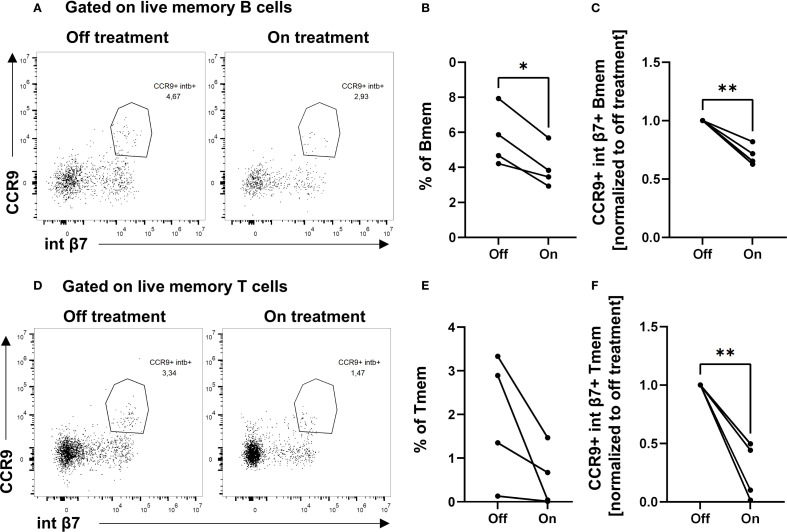
Decreased percentages of CCR9^+^ integrin β7^+^ double-positive memory B cells and memory T cells in patients on BXR treatment. PBMCs from cutaneous T-cell lymphoma patients (n = 4) on and off bexarotene (BXR) treatment were stained for the gut-homing markers CCR9 and integrin β7 (int β7). **(A, D)** The percentage of CCR9^+^ integrin β7^+^ memory B cells (Bmem) and memory T cells (Tmem) is shown as representative figures from one patient, as well as **(B)** the percentage of double-positive cells of memory B cells and **(E)** memory T cells, and **(C, F)** normalized to baseline values for each patient. PBMCs, peripheral blood mononuclear cells.

To validate the functional relevance of BXR on lymphocytes *in vivo*, PBMCs from CTCL patients with or without BXR treatment were analyzed using flow cytometry. The data showed CCR9^+^ integrin β7^+^ double-positive B memory cells (CD27^+^ IgD^−^) in the peripheral blood without BXR treatment (**Figures 5A–C**; mean off treatment: 5.7%). In contrast to the *in vitro* results, these cells were observed in lower frequencies in the blood on BXR treatment (mean: 4%, p = 0.01; **Figures 5A–C**). Similarly, the frequencies of integrin β7^+^ CCR9^+^ memory T cells (CD45RO^+^) decreased with BXR treatment (mean off: 1.9%, on: 0.55%; p = 0.009; **Figures 5D–F**). In line with a lack of negative impact of BXR on the viability, proliferation, and induction of gut-homing receptor expression, the observed lower frequencies of CCR9^+^ integrin β7^+^ B and T lymphocytes in CTCL patients treated with BXR suggest that these cells exited the bloodstream, potentially toward the gut or other barrier tissues.

## Discussion

In this study, we show that BXR induces CD38 and TGM2, and also the gut-homing receptors CCR9 and integrin β7 in T and B lymphocytes from healthy individuals *in vitro*, while suppressing the skin-homing molecule CLA. *Ex vivo* analysis of integrin β7^+^ and CCR9^+^ lymphocytes of CTCL patients under BXR treatment showed a lower frequency in the blood compared to patients without BXR treatment, suggesting increased gut homing. These findings on gene regulation with BXR are similar to those of other retinoids including ATRA or 9cRA on CD38 (B cells: 12), TGM2 (B cells: 12), CCR9 (B cells: 15, T cells: 14, 29), integrin β7 (B cells: 11, 15, T cells: 14, 29), and skin-homing markers (T cells: 14, 29). Since retinoids signal via different DNA motifs (termed RARE/DR-5) compared with rexinoids (RXRE/DR-1), we analyzed and confirmed the close proximity of RARE/DR-5 and RXRE/DR-1 in the promoters of the respective target genes, which could explain the similar effect at the molecular level. Of note, RARi also reduced BXR-driven RXR activation and the upregulation of CD38 and TGM2 in T and B cells. This may result from RAR–RXR heterodimerization after binding of the inhibitor as well as RXR. RXR activation alone in existing heterodimers is not transactive (30).

The direct signaling of BXR in B and T lymphocytes was validated at the protein level by the regulation of surface marker expression. Of note, the dose–response analysis suggests that an approximately 10-fold higher concentration of BXR is needed to induce the same effects as 9cRA on most of the investigated genes, including CD38. Similarly, Wang et al. demonstrated that 10-fold higher BXR concentrations compared with ATRA were required to induce comparable integrin β7 activation in CTCL cell lines (17). The differences observed in this study between the induction of BXR target genes in B and T cells are related to the baseline gene expression of each cell type. For example, the high baseline CD38 expression by T cells was only slightly upregulated by BXR, or TGM2 was expressed at very low levels in B cells, with regulation by BXR at mRNA and protein levels below the limit of detection.

In human B cells, it has not yet been reported that BXR upregulates the gut-homing markers CCR9, integrin α4, and integrin β7 with reduced skin-homing marker CLA. The findings are similar to observations from human B cells in response to ATRA (15), which were also confirmed to be relevant *in vivo.* A preclinical model of dominant negative RARα B cells in mice showed that when RA signaling was abrogated in B cells, the cells expressed less integrin α4β7 in the presence of RA (11). In human T cells from healthy donors, these homing receptors were regulated in a comparable fashion in response to ATRA (14). Also with BXR, integrin β7 was induced in CTCL cell lines, similar to ATRA (17), and also in murine T cells (16, 29). CCR9 and integrin α4β7 expression being controlled by BXR and retinoids in human and murine B and T cells in a comparable manner suggests a conserved mechanism supporting functional relevance. Of note, in this study, the reduced frequencies of CCR9^+^ and integrin β7^+^ memory B and T cells in the blood of CTCL patients treated with BXR suggest that these cells left the blood, e.g., by homing into barrier tissues such as the gut or lung. Supporting the hypothesis, BXR-stimulated CTCL cell lines also upregulated integrin β7 expression and adhered to the immobilized ligand, the mucosal vascular addressin cell adhesion molecule 1 (MAdCAM-1)-Fc (17). Also, splenic lymphocytes from integrin β7 knockout mice failed a MAdCAM-1-Fc migration assay or homing toward the small intestine and colon on adoptive transfer (31). Beyond, ATRA signaling upregulated CCR9 expression in murine CD4^+^ T lymphocytes leading to migration towards the c-chemokine ligand 25 (CCL25) (29) *in vitro* and into the small intestine on adoptive transfer (31).

CCR9^+^ and integrin β7^+^ memory B and T cells leave the blood of CTCL patients through a BXR-dependent program that promotes gut homing. This is further supported by our finding that BXR promotes plasmablast differentiation, similar to previous reports on 9cRA (12). Beyond the upregulation of classical differentiation markers such as CD38 and CD27, BXR increased the secretion of IgA and IgM by B cells on day 4, similar to other studies on 9cRA or ATRA stimulation (12, 28, 32). We observed increased proliferation, assessed using CFSE dilution, in activated B cells with BXR, a prerequisite of Ig isotype switch and plasmablast differentiation (33). These findings are in line with previous reports on activated B cells with ATRA in this setting (28). However, BXR was proposed as anti-proliferative in CTCL cell lines (34). However, an MTS assay instead of CFSE dilution was used, implying that mitochondrial functions, not proliferation, were assessed. Accordingly, the survival of T and B cells was not affected by BXR or 9cRA in our experiments, as also observed by others (17).

In conclusion, our data extend the current knowledge obtained from retinoids, including ATRA and 9cRA, that induce homing to the gut at the expense of the skin in human B and T lymphocytes by the rexinoid BXR. Understanding the physiological functions of RXR ligands as endogenous 9cRA on lymphocytes may contribute to better treatment of T cell- or B cell-driven diseases such as autoimmunity and allergies.

## Data Availability

The original contributions presented in the study are included in the article/**Supplementary Material**. Further inquiries can be directed to the corresponding author.
