# Supplementary material for: Bexarotene signaling in human B and T lymphocytes induces gut-homing receptor expression
Source: Front Immunol. 2025 Oct 8;16:1664199. doi: 10.3389/fimmu.2025.1664199 (PMC12540313; doi:10.3389/fimmu.2025.1664199)
Supplement: Supplementary file 1 [file DataSheet1.docx]

**Supplementary data:**

**Supplementary Table 1: Patient characteristics.** Overview of patient age, sex, disease stage and score as well as medication for n=4 cutaneous T cell lymphoma patients.

| Pat ID | Age | Sex | Disease stage | Clinical manifestation on BXR treatment | Medication |
| --- | --- | --- | --- | --- | --- |
| #1 | 57 | female | Mycosis fungoides T2bN0M0B0, stage IB | disseminated perifollicular maculae, plantar flat plaques, and 1 node, MSWAT 3 | Bexarotene 150 mg for 7 months;  UVB 311nm |
| #2 | 67 | female | Mycosis fungoides, T1bN0M0B0, stage IA | Upper trunk and both thighs with erythematous plaques, MSWAT 0.5 | Bexarotene 300 mg for >12 months;  topical mometasone |
| #3 | 58 | male | Mycosis fungoides, T1aN0M0B0, stage IA | Face with progressive plaques, MSWAT 0.5 | Bexarotene 300 mg for 4 weeks;  topical methylprednisolone-aceponate, |
| #4 | 43 | female | Mycosis fungoides T3bN0M0, stage IIB | Right upper arm and left upper leg with max. 1.5 cm nodes | Bexarotene 300 mg for 8 weeks;  topical triamcinolone |

**Supplementary Table 2: DR-1, DR-2 and DR-5 motifs in retinoid response genes CD38, TGM2, CCR9, integrin α4, integrin β7 and CLA.** Promoter analysis for DR-1, DR-2 and DR-5 motifs in retinoid response genes CD38, TGM2, CCR9, integrin α4, integrin β7 and CLA by comparing related motif profiles from the JASPAR database to genomic sequences downloaded from the UCSC genome browser. Highlighted (yellow): positive control: the reported DR-5 in the first intron of CD38 (Kishimoto et al. (1998); 22).

| **motif_id** | **motif_alt_id** | **Gene name** | **start** | **stop** | **strand** | **score** | **p-value** | **q-value** | **matched_sequence** | **spacing** |
| --- | --- | --- | --- | --- | --- | --- | --- | --- | --- | --- |
| MA0115.1 | MA0115.1.NR1H2::RXRA | CD38 | 15774218 | 15774234 | - | -1.85714 | 2.77E-05 | 0.548 | AGAAGTCCAAGATCAAG | DR-1 |
| MA0730.1 | MA0730.1.RARA | CD38 | 15775964 | 15775980 | - | 3.83607 | 5.60E-05 | 0.475 | AGGTCTGTCAAAGATCA | DR-1 |
| MA1148.2 | MA1148.2.PPARA::RXRA | CD38 | 15772297 | 15772313 | - | 10.5764 | 7.44E-05 | 0.601 | TTATAGTGCAAGGTTCA | DR-1 |
| MA0115.1 | MA0115.1.NR1H2::RXRA | CD38 | 15769204 | 15769220 | - | -5.79592 | 7.50E-05 | 0.665 | ACAGATCCAAGGTCTTC | DR-1 |
| MA0115.1 | MA0115.1.NR1H2::RXRA | CD38 Intron1 | 15792365 | 15792381 | - | 11.2347 | 7.16E-07 | 0.0975 | CAGGGTCATAGGACAAT | DR-1 |
| MA0115.1 | MA0115.1.NR1H2::RXRA | CD38 Intron1 | 15792107 | 15792123 | - | 8.61224 | 1.50E-06 | 0.0975 | TAAGGTCACAGATCAAC | DR-1 |
| MA0855.1 | MA0855.1.RXRB | CD38 Intron1 | 15792109 | 15792122 | - | 2.56364 | 4.21E-05 | 0.66 | AAGGTCACAGATCA | DR-1 |
| MA1148.1 | MA1148.1.PPARA::RXRA | CD38 Intron1 | 15783095 | 15783112 | - | 11.3333 | 4.56E-05 | 0.588 | ATCTTGGTCAGAAGGCAG | DR-1 |
| MA0115.1 | MA0115.1.NR1H2::RXRA | CD38 Intron1 | 15806771 | 15806787 | - | -6 | 7.84E-05 | 0.665 | TGGGCACAAATGTCAAG | DR-1 |
| MA0115.1 | MA0115.1.NR1H2::RXRA | CD38 Intron1 | 15802232 | 15802248 | + | -6.73469 | 9.42E-05 | 0.665 | agaggtgaaagatctct | DR-1 |
|  |  |  |  |  |  |  |  |  |  |  |
| MA0729.1 | MA0729.1.RARA | CD38 | 15770489 | 15770506 | + | 1.78788 | 5.82E-06 | 0.296 | gaggtcaggagttcatga | DR-2 |
| MA0729.1 | MA0729.1.RARA | CD38 | 15770056 | 15770073 | - | -1.45455 | 1.22E-05 | 0.296 | GAGGTCAAGAGTTCGAGA | DR-2 |
| MA0729.1 | MA0729.1.RARA | CD38 | 15774860 | 15774877 | + | -2.19697 | 1.44E-05 | 0.296 | gagctcagcaggtcaagg | DR-2 |
| MA0729.1 | MA0729.1.RARA | CD38 | 15774707 | 15774724 | + | -8.72727 | 5.59E-05 | 0.351 | gagtccaggagttcaaga | DR-2 |
| MA0729.1 | MA0729.1.RARA | CD38 Intron1 | 15780588 | 15780605 | - | 6.07576 | 2.02E-06 | 0.197 | AAGGTCAAGATTTCAGGG | DR-2 |
| MA0729.1 | MA0729.1.RARA | CD38 Intron1 | 15780050 | 15780067 | - | -2.98485 | 1.71E-05 | 0.296 | AAGGTGAAAATTTCATGA | DR-2 |
| MA0729.1 | MA0729.1.RARA | CD38 Intron1 | 15780893 | 15780910 | + | -5.45455 | 2.89E-05 | 0.296 | aaggtcaggagtacaaga | DR-2 |
|  |  |  |  |  |  |  |  |  |  |  |
| MA0159.1 | MA0159.1.RARA::RXRA | CD38 | 15768725 | 15768741 | - | 12.2449 | 2.02E-05 | 0.492 | GGTGGATCATGAGGTCA | DR-5 |
| MA1149.1 | MA1149.1.RARA::RXRG | CD38 | 15773494 | 15773511 | - | 10.9818 | 5.40E-05 | 0.481 | GGGGGCAAAAGAAGGGTG | DR-5 |
| MA1149.1 | MA1149.1.RARA::RXRG | CD38 Intron1 | 15779084 | 15779101 | - | 15.3818 | 2.91E-06 | 0.234 | GGGGGCACTTTCGGGTCA | DR-5 |
| MA0159.1 | MA0159.1.RARA::RXRA | CD38 Intron1 | 15784830 | 15784846 | + | 11.7143 | 2.67E-05 | 0.577 | ggtggatgacgaggtca | DR-5 |
| MA0730.1 | MA0730.1.RARA | CD38 Intron1 | 15781243 | 15781259 | + | 5.29508 | 3.57E-05 | 0.475 | aggacataatagggtct | DR-5 |
| MA1149.1 | MA1149.1.RARA::RXRG | CD38 Intron1 | 15791293 | 15791310 | + | 11.0909 | 5.08E-05 | 0.481 | aggggggagggggggtca | DR-5 |
| MA1149.2 | MA1149.2.RARA::RXRG | CD38 Intron1 | 15789784 | 15789800 | - | 10.7364 | 6.43E-05 | 0.53 | AGGGCATCACACGGTGA | DR-5 |
| MA0159.1 | MA0159.1.RARA::RXRA | CD38 Intron1 | 15808716 | 15808732 | + | 9.13265 | 9.09E-05 | 0.622 | agttcaaaatgatgtta | DR-5 |
|  |  |  |  |  |  |  |  |  |  |  |
| MA0855.1 | MA0855.1.RXRB | TGM2 | 38123545 | 38123558 | - | 16.5636 | 4.48E-07 | 0.0864 | AGGTTCAGAGGTCA | DR-1 |
| MA1148.1 | MA1148.1.PPARA::RXRA | TGM2 | 38123311 | 38123328 | - | 11.3819 | 4.42E-05 | 0.588 | AACAAGATTAAAGTTCAG | DR-1 |
|  |  |  |  |  |  |  |  |  |  |  |
| MA0729.1 | MA0729.1.RARA | TGM2 | 38118658 | 38118675 | - | -10.6061 | 8.04E-05 | 0.357 | ACTGTCAAACTGTCAATC | DR-2 |
|  |  |  |  |  |  |  |  |  |  |  |
| MA0159.1 | MA0159.1.RARA::RXRA | TGM2 | 38118947 | 38118963 | + | 16.9592 | 1.03E-06 | 0.1 | agttcagccagacgtca | DR-5 |
| MA1149.2 | MA1149.2.RARA::RXRG | TGM2 | 38125827 | 38125843 | + | 15.0182 | 3.87E-06 | 0.219 | ggggcaattacgggtca | DR-5 |
| MA1149.2 | MA1149.2.RARA::RXRG | TGM2 | 38126387 | 38126403 | + | 12.0455 | 2.97E-05 | 0.511 | aggtgatgcatgggtga | DR-5 |
| MA1149.2 | MA1149.2.RARA::RXRG | TGM2 | 38119354 | 38119370 | - | 11.8091 | 3.43E-05 | 0.511 | GTGTCAATGAAGGTTCA | DR-5 |
| MA0159.1 | MA0159.1.RARA::RXRA | TGM2 | 38123731 | 38123747 | - | 9.69388 | 7.07E-05 | 0.622 | GGTGGAGACAAAGGTGA | DR-5 |
|  |  |  |  |  |  |  |  |  |  |  |
| MA0115.1 | MA0115.1.NR1H2::RXRA | CCR9 | 45878933 | 45878949 | + | -2.17347 | 3.00E-05 | 0.548 | cgacatcataggtcatt | DR-1 |
| MA0115.1 | MA0115.1.NR1H2::RXRA | CCR9 | 45879470 | 45879486 | + | -2.7551 | 3.43E-05 | 0.548 | acagtaaaaaggtcagt | DR-1 |
|  |  |  |  |  |  |  |  |  |  |  |
| MA0729.1 | MA0729.1.RARA | CCR9 | 45881195 | 45881212 | - | 17.4394 | 7.08E-08 | 0.0138 | GAGGTCAAGAGTTCAGTG | DR-2 |
| MA0729.1 | MA0729.1.RARA | CCR9 | 45878863 | 45878880 | + | -5.40909 | 2.86E-05 | 0.296 | gaggtcaggagttcgaga | DR-2 |
|  |  |  |  |  |  |  |  |  |  |  |
| MA0159.1 | MA0159.1.RARA::RXRA | CCR9 | 45885143 | 45885159 | + | 14.7755 | 4.65E-06 | 0.181 | gggtgatgaaggggaca | DR-5 |
| MA0159.1 | MA0159.1.RARA::RXRA | CCR9 | 45876784 | 45876800 | + | 12.6837 | 1.60E-05 | 0.443 | gggtggtgaaaagttca | DR-5 |
| MA1149.1 | MA1149.1.RARA::RXRG | CCR9 | 45878449 | 45878466 | + | 12.4909 | 2.19E-05 | 0.39 | agggccagggacagggca | DR-5 |
| MA1149.2 | MA1149.2.RARA::RXRG | CCR9 | 45885052 | 45885068 | - | 10.7 | 6.56E-05 | 0.53 | AGGTCATCTGAAAGGTA | DR-5 |
| MA1149.2 | MA1149.2.RARA::RXRG | CCR9 | 45878490 | 45878506 | + | 10.2364 | 8.50E-05 | 0.629 | ggggcagggtgaggctg | DR-5 |
|  |  |  |  |  |  |  |  |  |  |  |
| MA1148.2 | MA1148.2.PPARA::RXRA | ITGA4 | 181452446 | 181452462 | - | 15.875 | 1.44E-06 | 0.103 | AATGGGGTCAAAGGGGA | DR-1 |
| MA1148.1 | MA1148.1.PPARA::RXRA | ITGA4 | 181448472 | 181448489 | + | 10.2292 | 9.22E-05 | 0.588 | gtttagatgagaagtcag | DR-1 |
| MA0115.1 | MA0115.1.NR1H2::RXRA | ITGA4 | 181454681 | 181454697 | + | -6.68367 | 9.33E-05 | 0.665 | caaggttagagatcact | DR-1 |
|  |  |  |  |  |  |  |  |  |  |  |
|  |  |  |  |  |  |  |  |  |  | no DR-2 |
|  |  |  |  |  |  |  |  |  |  |  |
| MA1149.1 | MA1149.1.RARA::RXRG | ITGA4 | 181455648 | 181455665 | - | 14.0909 | 7.57E-06 | 0.275 | GGGGGCATGGATGGGTTA | DR-5 |
|  |  |  |  |  |  |  |  |  |  |  |
| MA1148.1 | MA1148.1.PPARA::RXRA | ITGB7 | 53182124 | 53182141 | - | 16.9306 | 5.43E-07 | 0.105 | AAATGGGGCAAAGGTCTC | DR-1 |
| MA0856.1 | MA0856.1.RXRG | ITGB7 | 53187499 | 53187512 | + | 7.29787 | 1.45E-05 | 0.495 | gggtgcagagttca | DR-1 |
| MA1148.2 | MA1148.2.PPARA::RXRA | ITGB7 | 53183817 | 53183833 | + | 11.0069 | 5.65E-05 | 0.601 | ttataggtgatagggaa | DR-1 |
|  |  |  |  |  |  |  |  |  |  |  |
| MA0729.1 | MA0729.1.RARA | ITGB7 | 53188426 | 53188443 | - | -5.40909 | 2.86E-05 | 0.296 | GAGGTCAGGAGTTCGAGA | DR-2 |
| MA0729.1 | MA0729.1.RARA | ITGB7 | 53188881 | 53188898 | - | -5.40909 | 2.86E-05 | 0.296 | GAGGTCAGGAGTTCGAGA | DR-2 |
| MA0729.1 | MA0729.1.RARA | ITGB7 | 53184656 | 53184673 | - | -11.7121 | 9.90E-05 | 0.382 | CAGGTCATAGGGGCAGAG | DR-2 |
|  |  |  |  |  |  |  |  |  |  |  |
| MA1149.2 | MA1149.2.RARA::RXRG | ITGB7 | 53187401 | 53187417 | + | 19.0818 | 8.09E-08 | 0.0157 | aggtcaaggtgggggca | DR-5 |
| MA1149.2 | MA1149.2.RARA::RXRG | ITGB7 | 53183048 | 53183064 | + | 16.1818 | 1.52E-06 | 0.147 | aggtcaggcttggggca | DR-5 |
| MA0159.1 | MA0159.1.RARA::RXRA | ITGB7 | 53183715 | 53183731 | + | 15.398 | 3.10E-06 | 0.151 | gggtgacaggaaggaca | DR-5 |
| MA1149.2 | MA1149.2.RARA::RXRG | ITGB7 | 53187706 | 53187722 | - | 13.5545 | 1.11E-05 | 0.33 | GGGTCAGGGCGGGATCA | DR-5 |
| MA1149.1 | MA1149.1.RARA::RXRG | ITGB7 | 53183358 | 53183375 | - | 11.9909 | 2.98E-05 | 0.442 | GAGGTCAGGGAAGTGGGA | DR-5 |
| MA1149.1 | MA1149.1.RARA::RXRG | ITGB7 | 53189091 | 53189108 | - | 11.4091 | 4.22E-05 | 0.481 | AAGGTGATAGCAGGGTGG | DR-5 |
| MA0159.1 | MA0159.1.RARA::RXRA | ITGB7 | 53187561 | 53187577 | - | 9.77551 | 6.82E-05 | 0.622 | AGGGCAGGAGGAGCCCA | DR-5 |
| MA1149.1 | MA1149.1.RARA::RXRG | ITGB7 | 53183625 | 53183642 | - | 10.2182 | 8.29E-05 | 0.517 | GGGGGCGAGGGGGGGGTG | DR-5 |
| MA1149.1 | MA1149.1.RARA::RXRG | ITGB7 | 53186993 | 53187010 | - | 10.1818 | 8.46E-05 | 0.517 | AGGGGTGGGGAGAGGGCA | DR-5 |
| MA1149.1 | MA1149.1.RARA::RXRG | ITGB7 | 53182053 | 53182070 | - | 10.0545 | 9.06E-05 | 0.529 | GAGGCCACGAGAGGGGAA | DR-5 |
|  |  |  |  |  |  |  |  |  |  |  |
| MA1148.1 | MA1148.1.PPARA::RXRA | CLA | 108613449 | 108613466 | + | 15.875 | 1.45E-06 | 0.122 | aaatgggtaagagggcag | DR-1 |
| MA0856.1 | MA0856.1.RXRG | CLA | 108616540 | 108616553 | - | 8.85106 | 9.03E-06 | 0.495 | GGGCTCATAGGTCA | DR-1 |
| MA0115.1 | MA0115.1.NR1H2::RXRA | CLA | 108619660 | 108619676 | - | -2.33673 | 3.11E-05 | 0.548 | CGTGGTCATAGCTCACT | DR-1 |
| MA1148.1 | MA1148.1.PPARA::RXRA | CLA | 108613579 | 108613596 | + | 11.1875 | 5.02E-05 | 0.588 | cctgagggcagaagtcag | DR-1 |
| MA0855.1 | MA0855.1.RXRB | CLA | 108613452 | 108613465 | + | 1.61818 | 5.36E-05 | 0.66 | tgggtaagagggca | DR-1 |
| MA0856.1 | MA0856.1.RXRG | CLA | 108616927 | 108616940 | - | 0.914894 | 8.41E-05 | 0.667 | AAGGCCAGAGGGCA | DR-1 |
| MA0856.1 | MA0856.1.RXRG | CLA | 108613582 | 108613595 | + | 0.382979 | 9.60E-05 | 0.667 | gagggcagaagtca | DR-1 |
|  |  |  |  |  |  |  |  |  |  |  |
| MA0729.1 | MA0729.1.RARA | CLA | 108620649 | 108620666 | + | 0.575758 | 7.72E-06 | 0.296 | gaggtcgagagttcaaga | DR-2 |
| MA0729.1 | MA0729.1.RARA | CLA | 108615327 | 108615344 | - | -5.36364 | 2.83E-05 | 0.296 | GAGGTCAGGAGTTTAAGA | DR-2 |
| MA0159.1 | MA0159.1.RARA::RXRA | CLA | 108617787 | 108617803 | - | 9.17347 | 8.92E-05 | 0.622 | GGTTGGTCAACAGTACA | DR-2 |
|  |  |  |  |  |  |  |  |  |  |  |
| MA0159.1 | MA0159.1.RARA::RXRA | CLA | 108616529 | 108616545 | - | 17.051 | 9.66E-07 | 0.1 | AGGTCACCTGGAGTTGA | DR-5 |
| MA0730.1 | MA0730.1.RARA | CLA | 108618535 | 108618551 | + | 10.377 | 6.39E-06 | 0.34 | aggacaccctaggttca | DR-5 |
| MA1149.1 | MA1149.1.RARA::RXRG | CLA | 108616233 | 108616250 | - | 12.6818 | 1.94E-05 | 0.39 | GGGGTCAGGTGAAGTGCT | DR-5 |
| MA1149.1 | MA1149.1.RARA::RXRG | CLA | 108613490 | 108613507 | + | 11.2 | 4.77E-05 | 0.481 | aggggcagatagagggtg | DR-5 |
| MA0159.1 | MA0159.1.RARA::RXRA | CLA | 108615619 | 108615635 | - | 9.73469 | 6.94E-05 | 0.622 | GGGTTAGGAAGCTGTGA | DR-5 |

**
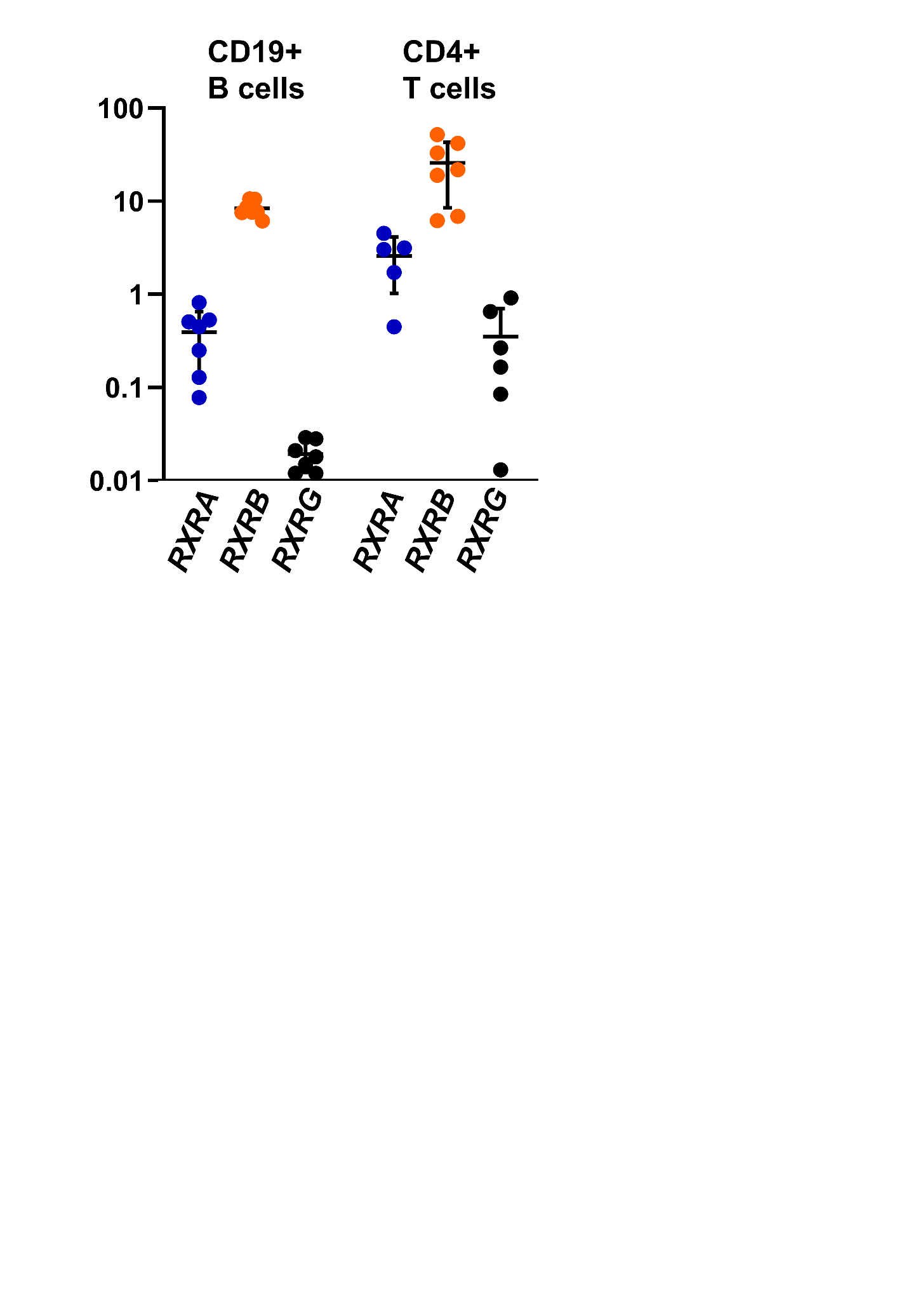
**

**Supplementary Figure 1. *RXRA*, *RXRB* and *RXRG* mRNA expression in activated CD19^+^ B cells and CD4^+^ T cells.** 2 days culture of activated CD19^+^ B cells (left, n=7) and CD4^+^ T cells (right, n= 5-7). The mRNA expression of RXRA (blue), RXRB (orange) and RXRG (black) was normalized to HPRT and each data represents one donor.


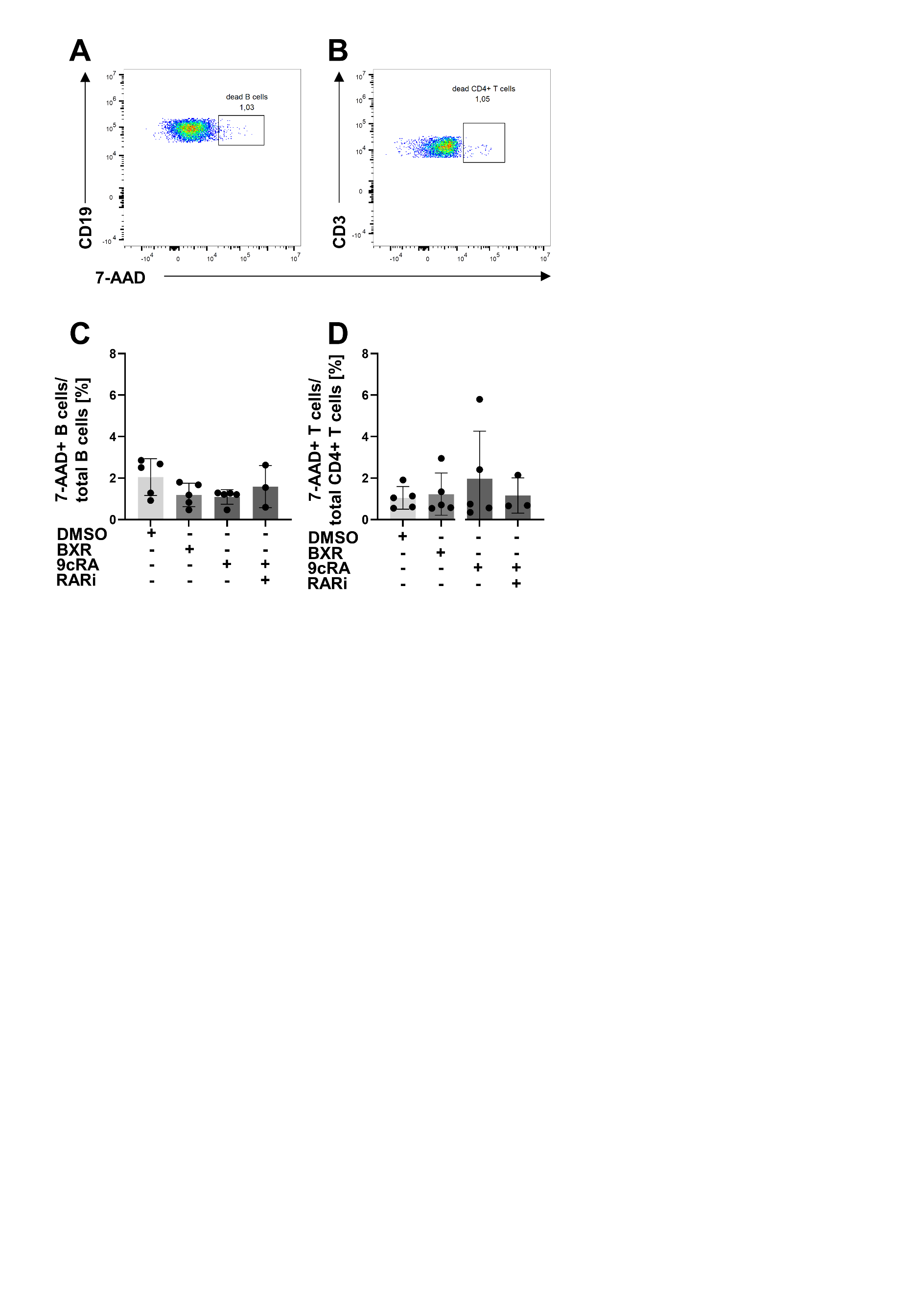


**Supplementary Figure 2. Bexarotene and 9-cis retinoic acid do not induce cell death in B and T cells.** Stimulation of **(A, C)** B cells and **(B, D)** T cells in presence or absence of BXR (1 µM) and 9cRA (0.1 µM) with or without the inhibitor RARi, analyzed for cell death by 7-AAD staining. Representative staining **(A, B)** and 7‑AAD positive cells comparing different stimulations **(C, D).** Each data point representing one healthy donor, error bars indicating mean+ SD.


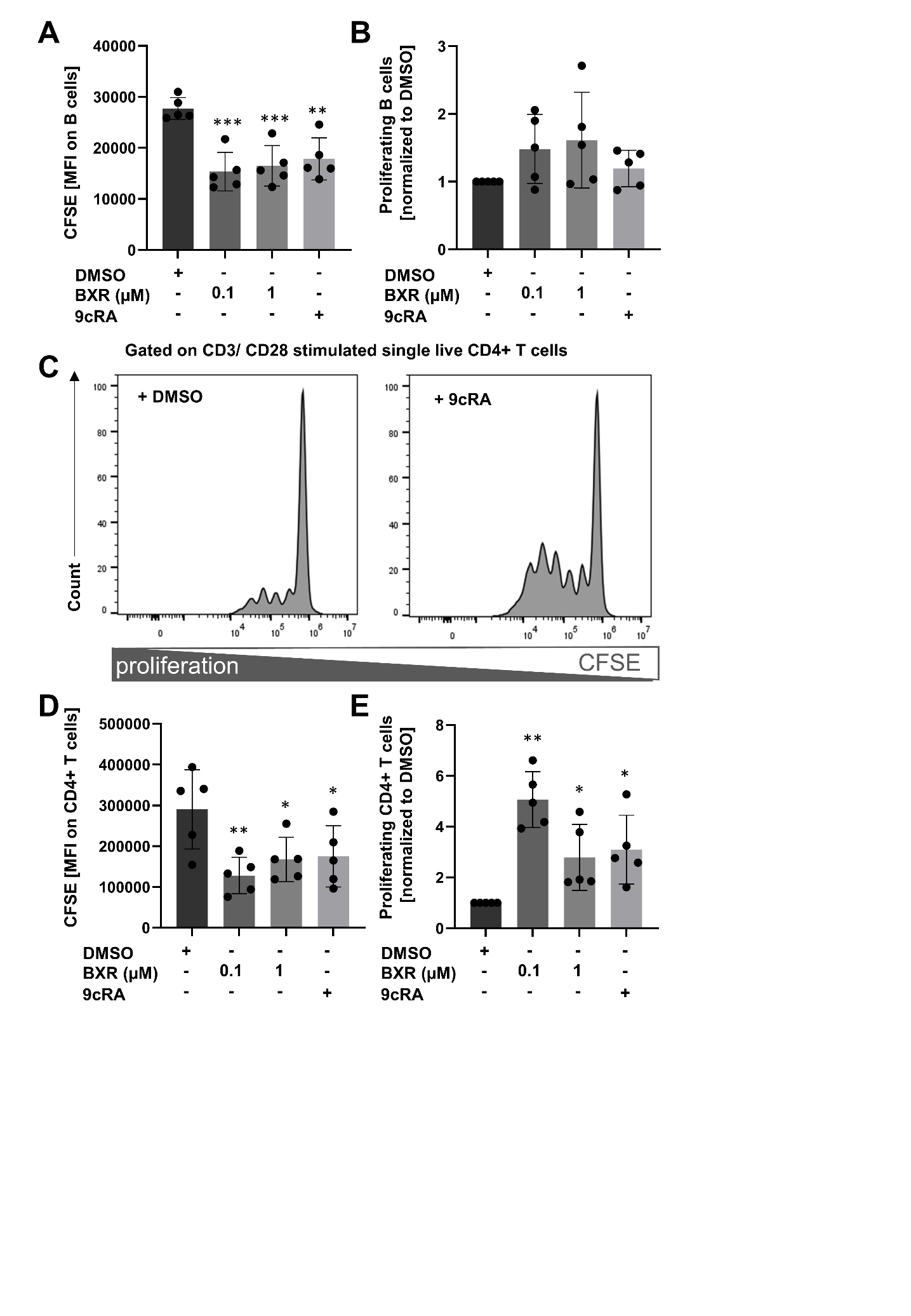


**Supplementary Figure 3. Bexarotene and 9-cis retinoic acid induce proliferation in B and T cells.** Stimulation of **(A, B)** B cells and **(C, D, E)** T cells in presence or absence of BXR (0.1 µM and 1 µM) and 9cRA (0.1 µM), analyzed as the CFSE MFI **(A, D)** and the normalized number of proliferating **(B)** B cells or **(E)** T cells**. (C)** Representative CFSE staining of stimulated T helper cells. Each data point representing one healthy donor, error bars indicating mean+ SD.
